# Supplementary material for: Splice-Junction-Based Mapping of Alternative Isoforms in the Human Proteome
Source: Cell Rep. Author manuscript; Available in PMC 2020 Jan 15. (PMC6961840; doi:10.1016/j.celrep.2019.11.026)
Supplement: 2 [file NIHMS1546469-supplement-2.zip › DF1/PXD006675/PulmonaryArtery/PulmonaryArtery_3_FLNA_FGGEHVPNSPFQVTAPER.pdf]

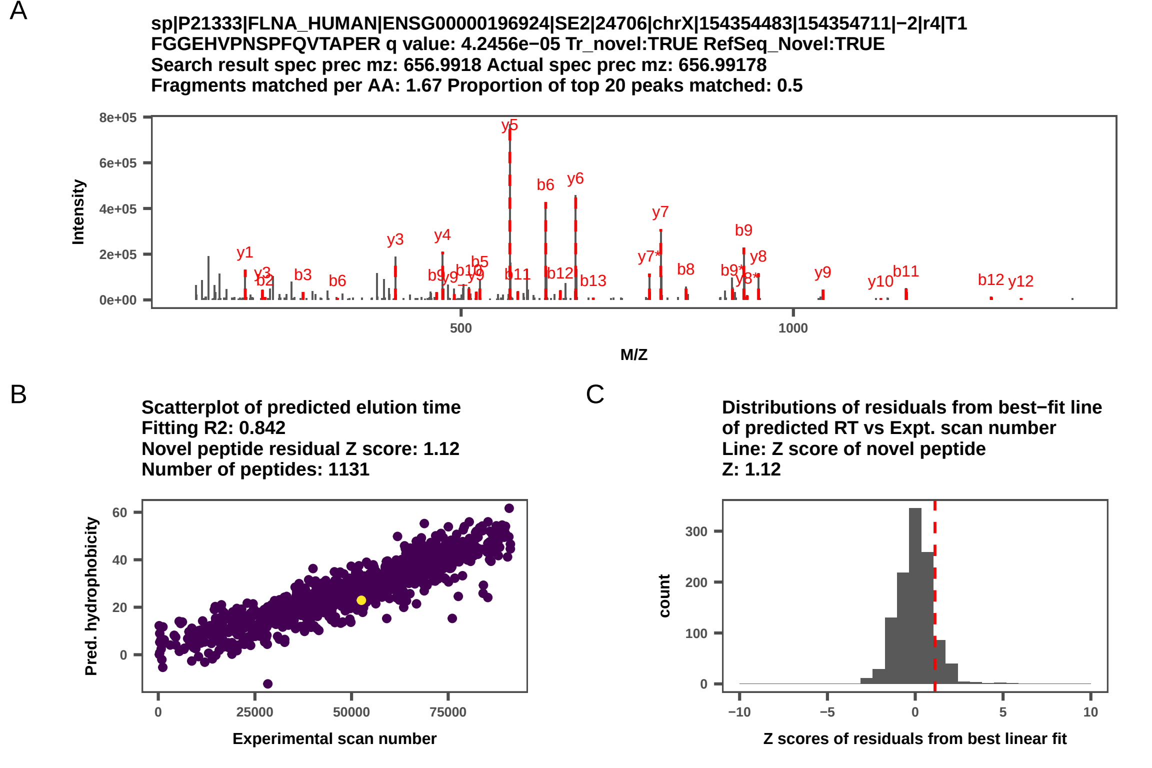
A
sp|P21333|FLNA_HUMAN|ENSG00000196924|SE2|24706|chrX|154354483|154354711|−2|r4|T1
FGGEHVPNSPFQVTAPER q value: 4.2456e−05 Tr_novel:TRUE RefSeq_Novel:TRUE
Search result spec prec mz: 656.9918 Actual spec prec mz: 656.99178
Fragments matched per AA: 1.67 Proportion of top 20 peaks matched: 0.5
Intensity
M/Z
B
Scatterplot of predicted elution time
Fitting R2: 0.842
Novel peptide residual Z score: 1.12
Number of peptides: 1131
Pred. hydrophobicity
Experimental scan number
C
Distributions of residuals from best−fit line
of predicted RT vs Expt. scan number
Line: Z score of novel peptide
Z: 1.12
count
Z scores of residuals from best linear fit
